# Report American Medical Association

**Published:** 1884-08

**Authors:** 


AMERICAN MEDICAL ASSOCIATION.

PHILADELPHIA, June, 1884.

*Dear Sir:*—At the meeting of the American Medical Association, held at Washington in May last, an Amendment to Regulation II. was adopted, which provides that—

*Membership in the Association shall be obtainable by any member of a State or County Medical Society recognized by the President and Secretary of said Society; and shall be retained so long as he shall remain in good standing in his local Society, and shall pay his annual dues to the Association.*

You will perceive that, as far as such opportunities are embraced, the strengh of the Association will be increased and consolidated, so as to unite the profession and give it a force and influence not otherwise attainable. Without undertaking, however, to point out the advantages of this action on the part of the Association, or to advocate the plan of which it is a main feature, it may simply be said that, as the new departure has been taken, it is for the Association and its constituent bodies to carry it out to the fullest extent, and to give the movement their hearty co-operation.

Toward this end, the first step is to make the action of the Association as widely known as possible; and you are, therefore, requested to bring the matter to the notice of your Society and its individual members, either by circular, or in such other way as may seem to you most effective for the purpose.

Applications for membership, in the manner specified above, accompanied with FIVE DOLLARS for annual dues, should be sent directly to the Treasurer, Dr. Richard J. Dunglison, Lock Box 1274, Philadelphia, Pa.; on receipt of which the weekly Journal of the Association will be forwarded for one year to such member. Respectfully yours,

WILLIAM B. ATKINSON, M. D.,
Permanent Secretary.

---

[Copies of this circular have been sent to the mayor (or presidents of village boards) of 644 cities, villages and towns in this State.]

ILLINOIS STATE BOARD OF HEALTH,
OFFICE OF THE SECRETARY,
SPRINGFIELD, July, 1884.

DEAR SIR:—At the recent meeting of the State Board of Health, held in Springfield, July 2d and 3d, 1884, the following resolution was adopted:

*Resolved*, That while epidemic cholera may be excluded from this country by thoroughly enforced quarantine regulations, yet the best attainable sanitary condition of every locality in the State should be secured, so that in the event of Asiatic cholera effecting an entrance, notwithstanding quarantine, the disease may be met and fought under the most favorable circumstances, and the Secretary is, therefore, hereby authorized to take such action as in his judgment will most promptly obtain a thorough sanitary organization of the State, and the adoption and enforcement of the measures necessary to improve its general sanitary condition.

It is entirely possible that we may escape a visitation of Asiatic cholera this year, although there is yet plenty of time for the disease to reach our shores before the cold weather. But even if there were no danger from this source, it should be remembered that everything which is done in the direction of sanitary improvement benefits the general health, reduces the amount of sickness, and lessens the death-rate. An obvious duty, therefore, rests at all times, but more urgently at present, upon those charged with the administration of public-health affairs to take such steps as may be necessary to remedy any defects in the existing sanitary status.

To this end a general inspection of the entire territory under your jurisdiction should be made forthwith; and all nuisances, or other conditions injurious to the public health, which may be disclosed by such inspection, should be promptly abated. Especial attention should be paid to—

*First*—The condition of the water-supply.

*Second*—The disposition of night-soil, garbage and sewage.

*Third*—The cleansing of streets, alleys, and other public places.

*Fourth*—The supervision of food-supplies, and of market-places, slaughter-houses and similar establishments.

*Fifth*—The general sanitation of every house and its surroundings.

1. Water is one of the commonest mediums through which cholera spreads; but, aside from this, typhoid and malarial fevers, diarrhea, dysentery and other diseases, are caused by impure and polluted water. Hence the necessity of protecting the supply from contamination by surface-washings and drainage of filthy soil or premises, or of water from manufacturing establishments, or by seepage through the ground from privy-vaults, cess-pools, etc.

2. Night-soil, garbage, sewage, and all other forms of decomposing organic matter, are highly prejudicial to health, and their foul odors are indications of danger. The various methods for their proper disposal, so as to render them harmless, are well understood, and should be enforced according to the varying conditions of each locality.

3. Clean streets and alleys, and gutters properly drained and kept free from unsightly and filthy accumulations, are of even greater importance during the heat of summer than at other times. The healthy condition of the atmosphere of a locality largely depends upon the condition of its thoroughfares.

4. The rapid decomposition of most articles of food during hot weather—the tainting, souring, wilting or rotting processes—and the derangements of the stomach and bowels caused by the use of such food, indicate the necessity for special supervision at this time of all food-supplies, and of the places where they are prepared, stored, or disposed of.

5. The foundation of healthy living is, obviously, the individual home and its surroundings. Houses, cellars, yards and out-buildings should be carefully inspected, and all accumulations of garbage, refuse and filth of every description should be removed, or, where this is not practicable, they should be rendered harmless by appropriate treatment. No house or premises can be healthy without proper drainage. If this cannot be secured by sewers or underground drains, then recourse should be had to surface drains, so as to prevent the possibility of stagnant water under the dwelling or its vicinity. Cellars should be dry, clean and well ventilated, so that they may not generate foul air to be drawn up through the house.

It is desired that this work of inspection, and remedying of evils and defects, be begun at the earliest practicable moment; and a preliminary report be made to this office, covering, in a general way, the existing sanitary condition, and the measures adopted and enforced for its improvement.

In connection with this report, information concerning your public-health provisions is also desired. I have, therefore, to request the names and addresses of your health commissioner, health officer, members of the board of health, or kindred officials; and copies of your health laws, ordinances, rules and regulations, etc.

With this information from every part of the State, the Board will be able to secure concert of action, and to direct, intelligently and efficiently, whatever measures may be found necessary should, unfortunately, any emergency arise requiring such action.

Forms of health ordinances, adapted to the various organizations of villages, towns and cities in the State, are now being prepared, and copies of the same will be furnished on application.

Confidently anticipating your early attention to this matter, in the interest of your community, I am,

Very respectfully,

JOHN H. RAUCH, *Secretary.*

